# Functional connections between bird eggshell stiffness and nest characteristics through risk of egg collision in nests

**DOI:** 10.1111/ele.14001

**Published:** 2022-03-15

**Authors:** Chih‐Ming Hung, Shu‐Han Tsao, Pei‐Lin Chiang, Shang‐Ping Wu, Mao‐Ning Tuanmu, Jia‐Yang Juang

**Affiliations:** ^1^ Biodiversity Research Center Academia Sinica Taipei Taiwan; ^2^ 33561 Department of Mechanical Engineering National Taiwan University Taipei Taiwan; ^3^ Thematic Center for Systematics and Biodiversity Informatics, Biodiversity Research Center Academia Sinica Taipei Taiwan; ^4^ 33561 Program in Nanoengineering and Nanoscience Graduate School of Advanced Technology National Taiwan University Taipei Taiwan

**Keywords:** avian evolution, eggshell strength, nest attachment, nest site, nest structure, passerine

## Abstract

Eggs and nests are two critical traits for the ecological success of birds. Their functional interactions, however, remain unclear. Here, we examined the functional connections between egg stiffness and nest attachment, site and structure for 1350 avian species. We revealed high eggshell stiffness for eggs in nests with a pensile attachment, located on non‐tree vegetation or having a domed shape, suggesting that birds produce stiffer eggs in response to higher egg‐collision risk in unstable or enclosed nests. Interdependence models suggested that the evolution of eggshell stiffness was more likely to be driven by than drive that of nest characters. Our results implied a trade‐off between investment in competing for established nesting niches and producing stiff eggs to explore novel niches with high collision risk, possibly mediated by predation or thermoregulation. This study highlights an overlooked connection between nests and eggshells that may have broadened the ecological niches of birds.

## Graphical abstract

This study establishes the form‐function connection between bird nests and eggs by examining the associations between eggshell stiffness and three nest characters for 1350 species. We provide empirical evidence that birds using unsteady or enclosed nests tend to produce eggs with high stiffness, revealing a long‐overlooked egg–nest interaction generated through the collision risk of eggs inside nests. The egg–nest interaction may also be associated with the evolution of birds and explain why birds can breed in diverse habitats.

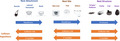

## INTRODUCTION

Novel traits may allow organisms to occupy various new ecological niches, leading to adaptive radiations (Stroud & Losos, 2016). For example, cleidoic eggs allowed early amniotes to leave the water and colonise diverse terrestrial habitats (Blackburn & Stewart, 2021; Sander, 2012). Birds can breed in a wide range of habitats, which is likely associated with the diverse characteristics of both their eggs and nests. Bird nests can protect eggs and create suitable microclimates for egg incubation (Deeming, 2016; Hansell, 2000). Therefore, many hypotheses on functional connections between nests and eggs have been proposed, especially between egg shape and nest site or structure (Birkhead et al., 2017, 2019, 2020; Duursma et al., 2018; Nagy et al., 2019; Stoddard et al., 2017; Tanaka et al., 2015). For example, the pyriform shape of some cliff‐nesting birds’ eggs is hypothesised to prevent eggs from rolling out of nests on cliff ledges (Gill, 1995; Hewitson, 1833), to avoid contamination by faeces, or to reduce mechanical pressure from incubating birds (Birkhead et al., 2017). However, our understanding of the correlated evolution between these two important life‐history traits of birds is still limited as evidence supporting those hypotheses has been found in only a few studies and for only a few species. So far only one Aves‐wide study has tested connections between egg shapes and nest locations or structure but does not find strong relationships (Stoddard et al., 2017), suggesting that some important and general mechanisms may be missing in existing hypotheses.

**FIGURE 1:**
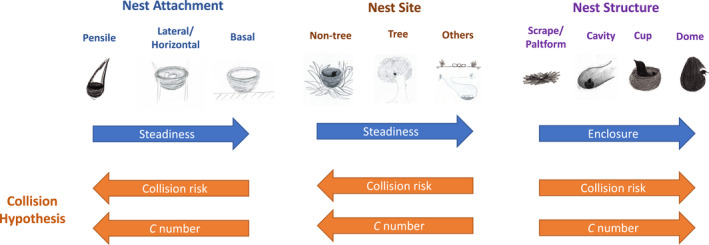
Predictions of the collision hypothesis tested in the study. Arrows indicate the direction from a low‐level/value to a high‐level/value. Nest sketches were generated by S.‐H.T.

Here, we proposed a new hypothesis explaining functional connections between egg and nest characteristics: the collision hypothesis, which predicts that eggs in a more unsteady or enclosed nest have evolved higher eggshell stiffness in response to higher collision risk in nests. Eggshells face two conflicting mechanical demands (Ar et al., 1979). First, eggs need to be strong enough to prevent themselves from being broken by colliding with other eggs or objects or being crushed by incubating birds. However, eggs cannot be too strong to be pipped by hatchlings, making them vulnerable to breakage, especially when the nests are moving (Board, 1982). Although nests are built to protect eggs, this protection may be compromised by accidental damage possibly related to nest steadiness and enclosure levels (see below for details), which are determined by what and how a nest is attached to and the structure of the nest (Figure 1).

Nests on unstable sites may suffer from nest damage due to wind (e.g. Rae & Rae, 2014; Rodgers Jr 1980), and eggs in an unstable, shaky nest are more likely to collide with one another than those in a stable nest (Kemal & Rothstein, 1988; Mallory et al., 2000). For example, studies on the behaviour of rejecting broken eggs show a lower rejection rate in birds building more stable nests, probably because eggs are subject to a higher risk of breakage in more unstable nests (Kemal & Rothstein, 1988; Mallory et al., 2000), such as those with loose attachment approaches (e.g. pensile attachment) or on moveable sites (e.g. reeds). Board (1982) argued that when parent birds are disturbed and escape from nests, their eggs face a high incidence of egg‐to‐egg collision in moving nests. Furthermore, eggs in enclosed nests (e.g. dome, cup or cavity nests) are presumably subject to higher risk of jostling other eggs than those in open, simple‐structured nests (e.g. platform nests) because their movement may be bounded by the enclosed nest structure. For example, Mallory and Weatherhead (1990) found that cavity‐nesting waterfowls tended to have thicker (stronger) eggshells than open‐nesting waterfowls and attributed this pattern to a higher risk of egg‐jostling in cavity nests than in platform nests when females enter or exist the nests. Surprisingly, it is uncommon to observe broken eggs in natural nests (e.g. egg‐breakage rates are 1%–8.5% for ducks, turkeys, falcons and penguins; Boersma et al., 2004), implying that cracked or broken eggs may be quickly rejected from the nests (Mallory et al., 2000) or that eggs and nests may have evolved together to overcome the egg‐cracking risk inside nests. It is thus expected that eggs facing higher collision risk associated with certain nest sites, attachment and structure types have evolved greater eggshell stiffness to reduce accidental damages from collision (Figure 1).

Eggshell strength has been studied thoroughly in the poultry industry because eggs may crack at the moment of lay, when being incubated or during collection or transportation (Nedomová et al., 2009; Solomon, 2010). However, only a few studies have examined how the eggshell strength of wild birds has evolved in response to breakage risk (Antonov et al., 2006; Igic et al., 2011), and even fewer have associated it with nest characteristics (Birkhead et al., 2017; Mallory & Weatherhead, 1990). Eggshell stiffness was found correlated with shell thickness squared (Juang et al., 2017). Although positive allometric relationships between shell thickness, egg mass and body mass explain a large part of the variation in eggshell stiffness among birds (Ar et al., 1979; Juang et al., 2017), there is still variation that may be caused by divergent selection forces, such as egg collision risk in nests, and is largely unexplored. A study argues that the vaterite coating on the eggshells of the Greater Ani (*Crotophaga major*) is an adaptation to the collision risk among eggs in nests (Portugal et al., 2018). Studies also suggest that brood parasitic birds, such as cuckoos and cowbirds, have evolved thicker and stronger eggshells to protect eggs from accidental breakage in hosts’ nests (Igic et al., 2011). The egg damage may result from the enlarged size of parasitised clutches, which increase the chance of jostling among eggs in nests, and from pecks by hosts or other parasitic birds (Blankespoor et al., 1982; Mermoz & Ornelas, 2004). However, correlated evolution between the characteristics of avian eggs and nests in response to collision risk among eggs inside nests spanning broad taxonomic groups remains unstudied.

To fill this knowledge gap, we tested whether eggs in a more unsteady or more enclosed nest have evolved higher eggshell stiffness for 1350 species from 37 orders across the avian phylogeny (Table S1). We treated nest unsteadiness and nest enclosure as two non‐mutually exclusive factors directly related to nest building and could explain the stiffness of eggs (Figure 1). Eggs in a more unsteady nest are assumed to face a higher egg‐to‐egg collision risk (Kemal & Rothstein, 1988; Mallory et al., 2000). We considered nests on the ground or cliff, located underground or piled up from the bottom of water are more stable than those on vegetation because the latter are more likely to swing in the wind or when the parent birds move on them. For the nests on vegetation, those located on non‐tree vegetation (e.g. reeds, leaves, vines or bushes) are less stable than those on trees because trees are generally more solid than other vegetation. Similarly, nests using pensile attachment are more likely to swing than those with other attachment types because the former are suspended in the air. Nests with lateral or horizontally forked attachment are more unsteady than those with support from the bottom. We thus expected that eggshell stiffness decreases as the level of nest steadiness presumably increases from the nests on non‐tree vegetation to those on trees and then on other sites, and from the nests with pensile attachment to lateral/horizontal and then basal attachment (Figure 1).

We also considered that eggs in more enclosed nests are more likely to hit one another because they tend to bounce inside the nests given the same level of nest unsteadiness (Mallory & Weatherhead, 1990). Dome and cup nests have an erected, surrounding rim (or wall), which are more enclosed than scrape/platform nests. Dome nests contain a smaller entrance (exit) than cup nests and thus are even more enclosed. Cavity nests do not always have a rim, which defines cup nests (see the definition in Supplementary Methods), and thus cavity nests on average have a lower enclosure level than cup nests. Therefore, we expected that eggshell stiffness decreases as the level of nest enclosure decreases from dome nests to cup, cavity and then scrape/platform nests. In addition, if eggs are subject to the risk of collision in nests, we expected that eggs in a larger clutch tend to have higher eggshell stiffness (Blankespoor et al., 1982; Mermoz & Ornelas, 2004). We thus assessed the associations between eggshell stiffness and three nest characters when the confounding effect of clutch size was controlled. Because egg mass and shape affect the absolute stiffness of eggshell (*K*), we used a dimensionless metric—*C* number—in this study to characterise relative eggshell stiffness (Juang et al., 2017). The *C* number was estimated from simulated *K* with respect to egg mass after removing geometry‐induced rigidity, allowing for comparing eggshell stiffness across taxonomic groups with a wide range of egg size and shape and even over the evolutionary history.

## MATERIALS AND METHODS

### Estimates of eggshell stiffness

We obtained egg images from the Arctos database (http://arctos.database.museum) of the Museum of Vertebrate Zoology at UC Berkeley and *The Book of Eggs* (Hauber, 2014). We then processed the images (Figure S1) using EGGXTRACTOR (Stoddard et al., 2017) to obtain the egg profile (see Supplementary Methods for details). To estimate eggshell stiffness, we used the finite element method (FEM), a numerical simulation method often used to solve engineering problems, with the commercial package ANSYS to perform compression simulations (Juang et al., 2017). The eggshell model was created using the egg profile obtained from the image processing and the thickness from the *Handbuch der Oologie* (Schönwetter & Meise, 1960). In the simulation, the modelled eggshell was meshed with a 4‐node structural shell element (SHELL181) and two circular compression plates (one on top of the eggshell, the other on the bottom) with a 20‐node structural solid element (SOLID186). The contact interface between the eggshell and plates was modelled by a 3D node‐to‐surface contact pair (CONTA175 and TARGE170) and was assumed frictionless. The bottom plate was fixed, and the top plate was restricted with a vertical degree of freedom only. The absolute stiffness (*K*) was obtained using a compression simulation resembling the actual compression test. The FEM estimates were verified by experimental and theoretical approaches (see Supplementary Methods for details).

Although the absolute stiffness of eggshells, *K*, can be accurately estimated by FEM, *K* is improper for interspecific comparison of egg resistance to external loads from other eggs or incubating birds due to the confounding effects of egg weight or geometry‐induced rigidity (see Supplementary Methods for details). To overcome this, Juang et al. (2017) developed a dimensionless metric, *C* number, to characterise eggs’ stiffness with respect to egg mass and shape. *C* number is defined as $C\equiv \frac{K}{W}\frac{A^{2}}{B}$, where *K* is the absolute stiffness (unit: N m^−1^; newton per meter) along the long axis; *A* and *B* are the breadth and length of the egg, respectively (unit: m); and *W* is the egg weight (unit: N). While *K* is determined by egg size and shape as well as eggshell thickness and Young's modulus, the *C* number removes those effects and represents relative stiffness with respect to the egg size. Note that the stiffness can alternatively be determined along the shell's short axis, $C\equiv \frac{K}{W}\left(\frac{2AB}{A+B}\right)$, and the value is similar to that obtained along the shell's long axis (Juang et al., 2017). In this study, we calculated the *C* number along the long axis as a measure of eggshell stiffness for 1350 species in 37 orders and 158 families (Table S1). We summarised the parameters used to estimate the *C* number and how they were determined in Table S2.

### Nest characteristics

We used three nest characters—site, structure and attachment—to categorise the nests of the 1350 species based on the descriptions on the *Handbook of the Birds of the World Alive* (del Hoyo, 2015), which is now the Birds of the World (https://birdsoftheworld.org/). Following the definitions in a previous study (Fang et al., 2018), we classified nests into six, five and four categories of nest site, structure and attachment respectively (Figure S2; see Supplementary Methods for the definition of each nest character category). We then reclassified the nest sites and attachment types according to nest stability and reclassified the nest structure types according to enclosure levels (as we presented in the introduction; Figure 1). When a species uses more than one type of nests and thus can be categorised into multiple stability or enclosure categories, we assigned the species to the category with the lowest stability or highest enclosure (i.e. highest collision risk); this is because these birds were assumed to have evolved eggs that can withstand the selective pressure associated with the highest risk. In addition, the ranking of nest unsteadiness and enclosure was intended to capture the general patterns among nest characters and thus is likely to be subject to a few exceptions. However, we aimed to test for broad‐scale patterns across avian species rather than individual‐specific patterns, so we believe that the broad ranking categories are sufficient for our purpose and a few exceptions are not likely to affect our findings.

### Egg stiffness differentiation among birds with different nest characteristics

We used phylogenetic generalised least squares (PGLS) models to examine the differences in eggshell stiffness among birds with different types of nest characters while considering the phylogenetic relationships among the species. To obtain information on the phylogenetic relationships, we extracted 1000 avian phylogenetic trees with the Hackett backbone of the 1350 species from https://birdtree.org/ (Jetz et al., 2012, 2014). We used 1000 trees to generate a majority‐rule consensus tree, using the least‐squares method to compute the edge lengths. We then built a PGLS model with the eggshell *C* number as the dependent variable and the reclassified categories of nest attachment, site or structure types as the independent variable, separately. We also built a single model including the types of all three nest characters as independent variables. We used Pagel's lambda model (Pagel, 1999) to estimate the error structure, and the parameter lambda was estimated using the maximum likelihood approach. Because clutch size had a significant effect on the eggshell *C* number after controlling for phylogenetic relationships among species (Table 1, Figure S3), we added clutch size as an additional independent variable in each PGLS model to account for the confounding effect. We obtained the clutch size for the 1350 species from MVZ, UC Berkeley (http://arctos.database.museum; each photograph of eggs represents one clutch, from which we also obtained egg images for *C* number estimation). We did not include the interaction between clutch size and nest types in the models because the interactive effect was not significant for all three nest characters. When the estimated coefficient for a nest character was significantly (*p* < 0.05) different from zero, we conducted multiple comparisons among the nest character categories using an AICc‐based model selection approach, which compared the PGLS models with all possible grouping patterns (Burnham et al., 2011; Dayton, 1998). We log‐transformed *C* number values before building the models.

**TABLE 1:** Summary of the PGLS models for examining the effects of (A) clutch size, (B) nest attachment, (C) nest site and (D) nest structure on *C* number among the studied species

	Coefficient	SE	t	*p*	Lambda
(A) Clutch size					0.494
Intercept	4.156	0.052	80.684	<0.001	
Clutch size	0.017	0.002	7.971	<0.001	
(B) Nest attachment					0.461
Intercept	4.144	0.049	85.231	<0.001	
Lateral/horizontal versus basal	0.052	0.019	2.719	0.007	
Pensile versus basal	0.111	0.024	4.551	<0.001	
Clutch size	0.017	0.002	8.197	<0.001	
(C) Nest site					0.444
Intercept	4.099	0.047	86.479	<0.001	
Tree versus others	0.050	0.011	4.697	<0.001	
Non‐tree vegetation versus others	0.081	0.010	7.759	<0.001	
Clutch size	0.017	0.002	8.098	<0.001	
(D) Nest structure					0.456
Intercept	4.099	0.046	89.696	<0.001	
Cavity versus scrape/platform	0.066	0.013	5.159	<0.001	
Cup versus scrape/platform	0.070	0.011	6.619	<0.001	
Dome versus scrape/platform	0.136	0.014	9.572	<0.001	
Clutch size	0.016	0.002	7.586	<0.001	

Note

In the models for nest characters, clutch size was included as an independent variable to account for its confounding effect.

We further examined the potential confounding effect between nest attachment and site, both of which determine the unsteadiness of a nest on the *C* number. Thus, we built another PGLS model to include nest attachment, nest site and their interaction as the independent variables and the eggshell *C* number as the dependent variable. However, we could not build a model that included every nest attachment and site type because some nest attachment and site types combinations did not exist among the 1350 species. Therefore, we further aggregated the categories into basal and non‐basal types for nest attachment and into non‐tree vegetation and others for nest sites in the PGLS analysis. This section's analyses were conducted in R with the *phytools*, *nlme*, *ape* and *AICcmodavg* packages.

### Evolutionary interdependence between nest characters and eggshell stiffness

We examined the interdependence between the evolution of nest character types and that of eggshell stiffness using BayesTraits V3.0.1 (available: http://www.evolution.rdg.ac.uk/BayesTraitsV3.0.1/BayesTraitsV3.0.1.html; Pagel et al., 2004). We built two models, one assuming that a nest character and eggshell stiffness have evolved independently and the other assuming that they have evolved in a correlated fashion, using the Markov Chain Monte Carlo (MCMC) method to estimate transition rates between characters types over evolution. We then compared the independent to the dependent models using Bayes Factors. Since the model approach requires two binary characters, we reclassified *C* number values into ‘high’ and ‘low’ categories using the median value among the 1350 species as cut‐off. We also reclassified nest attachment into ‘basal’ and ‘non‐basal’ categories, nest sites into ‘tree or non‐tree vegetation’ and ‘others’ categories, and nest structure into ‘scrape/platform’ and ‘others’ categories. We ran an MCMC chain with 5.05 million iterations and a burn‐in of 50,000 iterations and sampled every 1000 iterations. We scaled the branch length of the phylogenetic trees by 0.001 and used an exponential prior with a mean of 10 for all parameters.

### Ancestral state reconstruction and examination of *C* number changes across avian evolution

We estimated the *C* number of internal nodes (i.e. ancestral states) in phylogenies using BayesTraits to examine how the *C* number of avian eggs has evolved over time. We used the Random Walk model, which assumes non‐directional evolution, and the MCMC method to estimate the values based on the 1000 phylogenetic trees. We ran several runs of the model with different setting parameters—including different priors, iterations, burn‐ins and sample periods—and estimated the averages and standard deviations of estimated parameters to make sure that the estimation converged (Figure S4). In the end, the analysis was performed with an exponential prior with a mean of 0.001, an MCMC chain of 500,000 iterations, and a burn‐in of 200,000 iterations and sampled every 1000 iterations. We estimated the *C* number for each of the 1201 internal nodes, identified using BayesTrees V1.3 (available from: http://www.evolution.rdg.ac.uk/BayesTrees.html). Not all trees had the same internal nodes, so we used the Most Recent Common Ancestor (MRCA) approach in BayesTraits. For some trees, the nodes might include other species aside from the tip labels we defined, but all trees were considered.

To analyse the trend in *C* number over time, we used the quantile regression to examine the relationship between the median *C* numbers of tips (i.e. extant species) and nodes (i.e. ancestral species) and their node depths. The node depths were the edge lengths from the nodes or tips to the root of the phylogenetic tree, indicating their relative lengths of evolutionary times. Furthermore, we also included a binary variable, indicating whether a tip or a node was a passerine species or not, and its interaction with the node depth as additional predictors in a quantile regression model to examine the difference in *C* number changes between passerines and non‐passerines across the evolution of birds. To visualise the non‐linear trends in *C* number changes over time, we fitted the values with polynomial spline curves. The quantile regression was conducted with the *quantreg* package in R, and a bootstrap approach was used to estimate the standard error of the coefficients in the models.

## RESULTS

### Eggshell stiffness is strongly correlated with nest character types

**FIGURE 2:**
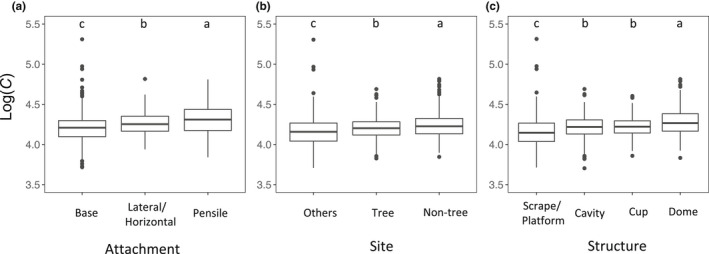
The eggshell *C* number of the birds using nests with different types of (a) attachment, (b) site and (c) structure after the effects of clutch size and phylogenetic relatedness among species being controlled. The values shown in the Figure 2 are the partial residuals for the nest character group from the phylogenetic generalised least squares models with nest characters and clutch size as independent variables. Boxplots show the median, interquartile range (IQR), extreme values up to 1.5 × IQR and outliers. Multiple comparisons between nest character groups were conducted using an AICc‐based model selection approach, which compares models with all possible grouping patterns. The alphabetical letter above each boxplot shows the grouping pattern with the lowest AICc value, with different letters indicating different groups. The *C* number was log‐transformed in the analysis

We found that values of the *C* number varied from 4776 to 233,377 (with the 5th and 95th percentiles being 8847 and 29,725 respectively) among the 1350 studied species. The result suggested that varying levels of egg stiffness may have evolved in response to divergent selection forces, such as collision risk, or simply resulted from genetic drift. The comparison of *C* numbers among birds with different nest character types showed consistent patterns with all three predictions of the collision hypothesis (Figures 1 and 2). Specifically, the eggshell *C* number increased from the birds using nests basally attached to those using nests with lateral/horizontal attachment and then those using nests with pensile attachment (Figure 2a). Similarly, the *C* number increased from the birds nesting on immovable objects, such as the ground, underground and cliffs, to those nesting on trees and then those nesting on non‐tree vegetation (Figure 2b). Finally, we also found that the eggshell *C* number was higher in the birds using dome nests than in those using cup or cavity nests and then in those using scrape/platform nests (Figure 2c). Thus, the results of PGLS models and multiple comparisons imply that birds using nests attached more unsteadily to objects, at more unstable sites, or with more enclosed structures (i.e. nests associated with higher egg collision risk) tend to have eggs with a higher *C* number—indicating higher stiffness (Figure 2 and Table 1). In addition, a single PGLS model including all three nest characters showed very similar results (Figure S5 and Table S3). The consistency between the observed and predicted associations suggests that collision risk associated with the nest characteristics is likely a driving force for the evolution of eggshell stiffness across the avian phylogeny.

We also found a significantly positive effect of clutch size on *C* numbers among the studied species in all PGLS models (Table 1). This provides additional evidence of the evolution of eggshell stiffness in response to collision risk among eggs in nests because a higher collision risk of eggs is expected in a larger clutch (Blankespoor et al., 1982; Mermoz & Ornelas, 2004). Finally, our analysis on the interactive effect of nest attachment and site showed that both non‐basal attachment types and non‐tree vegetation sites had significantly positive effects on *C* numbers without any significant interactive effect (Table S4). This result suggests that the two nest characters affected *C* numbers without confounding each other.

### Eggshell stiffness and nest characteristics are associated along avian evolutionary history

**FIGURE 3:**
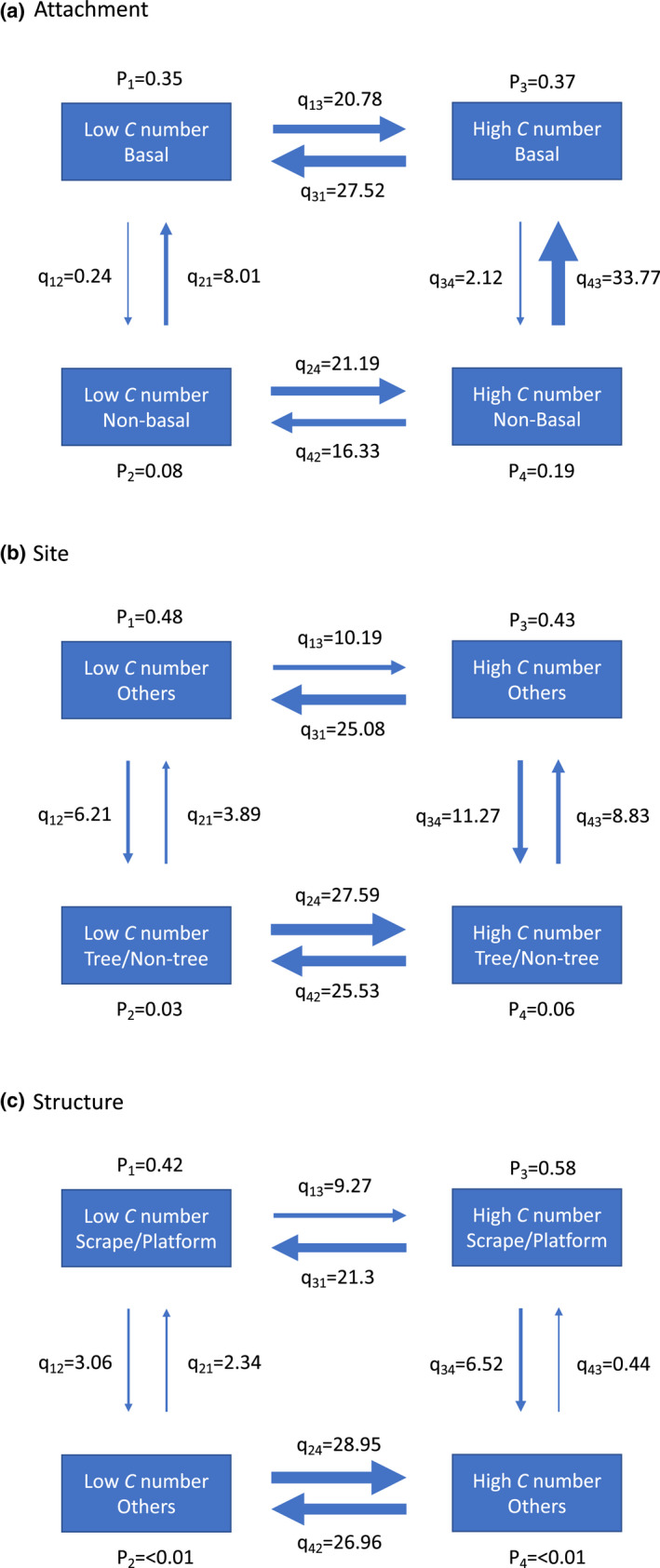
Evolutionary interdependence between the eggshell *C* number and (a) nest attachment, (b) nest site or (c) nest structure. For each nest character, the transition rates (q_ij_) between four different combinations of the *C* number and nest character categories were estimated by the dependent model in BayesTraits with the assumption that the two characters evolved interdependently. The thickness of the arrows is proportional to the estimated transition rates. The estimated probabilities of different character combinations are also shown for the root of the phylogenetic tree (p_i_)

The evolutionary interdependence analyses showed stronger support for dependent models than independent models between eggshell *C* numbers and either of nest attachment, site or structure (Bayes Factors = 9.3, 14.0 and 15.6 respectively). This indicated that the evolution of eggshell stiffness depended on the nest character types or vice versa. The dependent models showed that the eggshell *C* number of the birds using basal attachment, occupying nest sites other than trees or non‐tree vegetation, or using scrape/platform nests was more likely to transit from high to low values than vice versa. In contrast, the *C* number of the birds using non‐basal attachment, occupying non‐tree vegetation or tree nest sites, or using dome, cup or cavity nests was more likely to transit from low to high values than vice versa (Figure 3). These results suggested that higher eggshell stiffness was more likely to evolve in the birds using presumably more unsteady or more enclosed nests associated with higher collision risk. This may explain the differences in *C* number among extant birds with different nest character types (Figure 2). On the other hand, birds with either high or low *C* number values were more likely to transit from non‐basal to basal attachment, from other nest sites to tree/non‐tree vegetation sites, or from scrape/platform to other structure types than vice versa (Figure 3). This suggested that the evolution of nest character types may not strongly depend on the *C* number values and may explain the prevalence of basal attachment, tree/non‐tree vegetation sites and cup/cavity/dome nests in extant birds. Furthermore, the overall patterns of the dependent models also suggested that the evolution of *C* number was more likely to be driven by than drive that of nest characters (see the Supplementary Results for more details).

**FIGURE 4:**
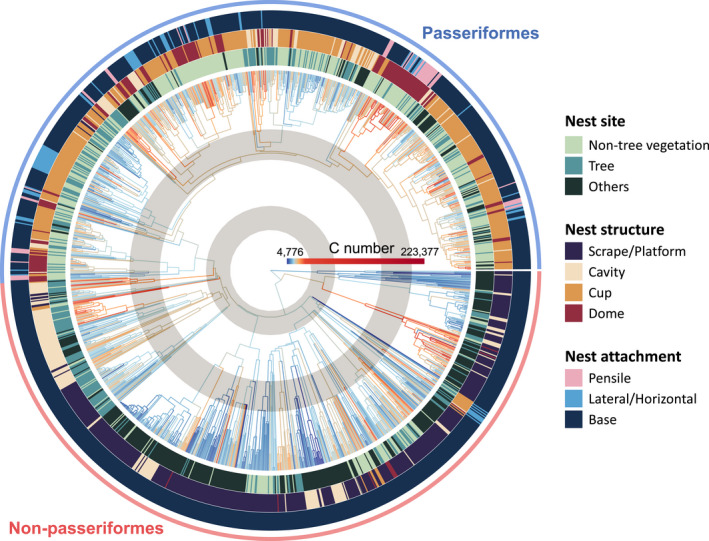
Estimated ancestral eggshell *C* numbers for the 1350 avian species studied. The colour of each branch of the phylogenetic tree shows the transition in estimated *C* number values from the rootward node to the tipward node or the tip. The types of nest site, nest structure and nest attachment of each species are shown at tips of the phylogenetic tree. The two grey rings indicate the two major adaptive radiation events in modern bird evolution. The colours in the outermost circle indicate whether a species is a passerine or not

**FIGURE 5:**
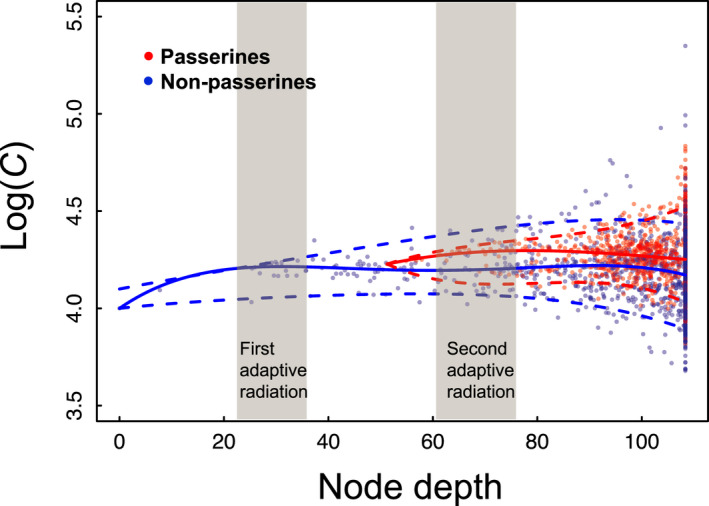
Eggshell *C* numbers of the 1350 avian species studied and the estimated values for their ancestors with separate evolution trends in passerine and non‐passerine birds along the avian phylogenetic tree. The blue solid and two blue dashed lines fit to locally polynomial spline curves for the medians and 5th and 95th percentiles of non‐passerine egg *C* numbers respectively; the red lines are for passerines. The two grey bands indicate the two major adaptive radiation events in modern bird evolution

We investigated the evolution of eggshell stiffness across the avian phylogeny (Figure 4) and analysed the trend of *C* number values along the avian evolution history (Figure 5). We found that the eggshell stiffness has evolved to increase in some lineages, but decrease in others (Figure 4). However, the depth of the nodes in the phylogenetic tree had no significant effect on the median values of *C* number (Table S5), suggesting that median eggshell stiffness has generally remained the same over the evolution of modern birds. Nevertheless, comparing the trend between passerines and non‐passerines, we found that both experienced reduction in eggshell stiffness over their evolutions, as the node depth showed a negative effect on *C* numbers without confounding effect from the passerine status (Table S5). The inconsistent trends found between all birds and passerines or non‐passerines may be due to non‐linear changes in *C* number over time. Polynomial spline curves fitted to the *C* numbers showed an early increase in eggshell stiffness followed by a gradual decline in passerines (Figure 5). In addition, the significantly positive coefficient of the passerine status in the model indicated that passerines had stiffer eggshells than did non‐passerines (Table S5), especially after the early stages of passerine evolution (Figure 5) when their use of pensile attachment, non‐tree vegetation sites and dome‐shaped nests increased (Fang et al., 2018). Therefore, the rise of passerines, which occurred when non‐passerines started to show reduced eggshell stiffness during their middle evolutionary stage, led to the overall non‐significant trend in median eggshell *C* numbers across the evolution of all modern birds.

## DISCUSSION

This study provides empirical evidence supporting the collision hypothesis, which suggests that an egg's resistance to collision inside a nest may be an important trait behind the correlated evolution between eggshells and nests. The evolutionary interdependence between eggshell stiffness and nest characters may also be associated with the evolution of birds. In their early stage of evolution, modern birds largely used scrape/platform or cavity nests on the ground, underground or on cliff/bank or trees with basal attachment (Fang et al., 2018); these birds also had relatively low eggshell *C* numbers (Figure 5). Given that passerines diverged more recently than non‐passerines, the ability to utilise unoccupied nest niches was critical to their survival. Passerines indeed evolved to use new nest attachment, site and structure types (i.e. pensile attachment, non‐tree vegetation and dome nests respectively; Fang et al., 2018), likely contributing to the increase in their eggshell stiffness. The pensile attachment, used only by passerines (Figure 4), allows nests to be built at novel nest sites, such as vines or leaves (mostly non‐tree vegetation; Fang et al., 2018). The lateral/horizontal attachment, which mainly occurs in passerines (Figure 4), may also facilitate the exploration of new nest sites. In addition, passerines used more dome and cup nests and fewer scrape/platform nests than did non‐passerines (Figure 4). However, pensile and lateral/horizontal attachment, non‐tree vegetation sites and dome/cup structure make nests presumably unsteady or enclosed, rendering eggs vulnerable to collision inside the nests (Board, 1982; Kemal & Rothstein, 1988; Mallory & Weatherhead, 1990). Consequently, passerines need to produce stiffer eggshells to increase their resistance to collision—indicated by the higher *C* number—a hypothesis supported by our results. Such egg–nest interactions might contribute to one major adaptive radiation (i.e. the second and also the largest one) in birds (Figure 5) that resulted in the explosive evolution of passerines that occupy diverse ecological niches (Barker et al., 2004; Prum et al., 2015).

There are trade‐offs between benefits and costs associated with increased eggshell stiffness. It has been known that eggshell needs to be as strong as possible to prevent the egg from cracking during incubation while still allowing the chick to hatch. Here, we further argue that the protective benefits of stiffer eggshells come with energetic costs. When controlling for egg shape and size, a higher *C* number corresponds to a thicker shell and/or a larger Young's modulus of the shell, indicating a higher level of investment in eggshells (see Supplementary Methods for details); this investment requires calcium, which is a critical but limited resource for reproduction in birds (Tilgar et al., 2002), and thus is energy expensive. Consequently, the alternative evolutionary strategies of (1) producing stiffer eggs to adapt to inferior (e.g. unstable) nest niches and (2) competing for better nest niches without spending extra energy to increase eggshell stiffness may be taken by different species or lineages over the avian evolution. It should be noted that factors other than niche competition, such as predation and thermoregulation, may also affect nest niche partitioning (Martin, 1988, 1996; Martin et al., 2017), which in turn mediates the interactions between nest characteristics and egg stiffness. For example, birds with smaller body sizes such as passerines are more vulnerable to predation and also more likely to be light enough to use nests that are attached to unstable nest sites (e.g. nests hanged down from vines), which are less accessible to predators (Collias & Collias, 1984). Thermoregulation is another challenge for nesting birds, especially for small species because of their high surface‐to‐volume ratios (Calder, 1984). Enclosed nests (e.g. dome nests) could provide additional benefits to retain heat and protect against rain or sun (Martin et al., 2017) at the expense of increased risk of egg collision. Thus, the energy costs of producing stronger eggs may also be outweighed by lower predation risk in less stable nests or greater thermal benefits in more enclosed nests.

This study helps establish the form‐function connection between bird nests and eggs from a novel perspective, explaining why birds can breed in diverse habitats. We examined egg and nest characters across the avian phylogeny and uncovered a possible evolutionary response of eggshell stiffness to the collision risk of eggs inside nests. This connection warrants further manipulative experiments across species in both laboratories and the field. Furthermore, our results suggest that some species, especially passerines, may have maximised their breeding niches by increasing eggshell stiffness to occupy novel nest niches, whereas others may have taken an alternative strategy to compete for stable nest niches. In addition, the interactions between eggs and nests may also be mediated by the predation risk and thermal benefits associated with diverse nest niches. Together, our findings demonstrate that the egg–nest interactions can also incur fitness costs while beneficial in some respects. These trade‐offs may explain the eggshell stiffness variation and diverse ecological niches of birds.

## AUTHOR CONTRIBUTION

C.‐M.H., M.‐N.T. and J.‐Y.J. conceived the study and supervised the project. S.‐H.T. led the data collection and analyses. P.‐L.C and S.‐P.W. contributed to data analyses. C.‐M.H., M.‐N.T, S.‐H.T. and J.‐Y.J. wrote the manuscript. All authors approved the final manuscript.

### PEER REVIEW

The peer review history for this article is available at https://publons.com/publon/10.1111/ele.14001.

## Supporting information

Table S1

Supplementary Material

## Data Availability

Data and code used in the study are available in the FigShare repository (https://doi.org/10.6084/m9.figshare.19295609).
