# Supplementary material for: Functional connections between bird eggshell stiffness and nest characteristics through risk of egg collision in nests
Source: Ecol Lett. 2022 Mar 15;25(6):1421–31. doi: 10.1111/ele.14001 (PMC9315002; doi:10.1111/ele.14001)
Supplement: Supplementary file 2 — Supplementary Material [file ELE-25-1421-s002.pdf]

Supplementary Information

Functional connections between bird eggshell stiffness and nest characteristics through risk of egg collision in nests

Chih-Ming Hung[1]†, Shu-Han Tsao[2]†, Pei-Lin Chiang[2], Shang-Ping Wu[2], Mao-Ning Tuanmu[1,3]* & Jia-Yang Juang[2,4]*

[1]Biodiversity Research Center, Academia Sinica, Taipei, Taiwan.

[2]Department of Mechanical Engineering, National Taiwan University, Taipei, Taiwan.

[3]Thematic Center for Systematics and Biodiversity Informatics, Biodiversity Research Center, Academia Sinica, Taipei, Taiwan.

[4]Program in Nanoengineering and Nanoscience, Graduate School of Advanced Technology, National Taiwan University, Taipei, Taiwan.

*Correspondence to:

mntuanmu@gate.sinica.edu.tw (M.-N. T.);

jiayang@ntu.edu.tw (J.-Y. J.).

†These authors contributed equally to this work.

Supplementary Methods

Supplementary Results

Supplementary Tables

Table S1. Bird species and their nest and egg traits examined in the study. (in a separate Excel file)

Table S2. Terminology and nomenclature in eggshell stiffness estimation.

Table S3. Summary of a single PGLS model for examining the effects of the three nest characters on *C* number among the studied species.

Table S4. Summary of the PGLS model for examining the interactive effect of nest attachment and site.

Table S5. Quantile regressions on the median of *C* number values for the 1,350 bird species studied and their ancestors.

Supplementary Figures

Figure S1. The image processing process.

Figure S2. Demonstration of various nest sites, structures, and attachment.

Figure S3. Relationship between clutch size and *C* number among the 1,350 bird species studied.

Figure S4. Convergence test results for BayesTraits analysis.

Figure S5. Variations in the eggshell *C* number values among birds using nests with different character types.

Supplementary References

Supplementary Methods

Egg images processing

The images obtained from the Arctos database (http://arctos.database.museum) of the Museum of Vertebrate Zoology at UC Berkeley and *The Book of Eggs* (Hauber 2014) were initially 8-bit RGB in JPEG format, and were transformed into greyscale images with the pixel value estimated using the following formula: $P = 0.299R + 0.587G + 0.114B$. After removing the salt and pepper noise with low-pass and median filters, the images were turned into binary images using Otsu's method (Otsu 1979) to contain only black and white. To make sure that the white area of the converted egg images by Otsu's method is the shape of an egg, we checked (i) the number of pixels of contiguous white to exclude the small white stains in the background of the image; (ii) a shape factor of the white area, defined as $f_{\mathrm{shape}} = 4\pi A / p^2$, where A stands for the number of pixels and p the perimeter of the white area—the shape factor is 1 for circles and 0.78 for rectangles; and (iii) the eccentricity of the white area (distance between the two foci divided by the length of the long axis) —eccentricity is 0 for circles and 1 for rectangles. We then only retained images that have (i) the number of pixels of contiguous white > 300, (ii) a shape factor > 0.86, and (iii) the eccentricity > 0.1. To ensure that the sharp poles faced upwards, we estimated the second moment of inertia of an oval whose value was identical to the egg contour. That is, the long axis of the oval was the same as that of the egg, and hence the angle between the y-axis and the long axis of the oval was the angle to rotate for the egg profile. Then we divided the right half of the egg into five parts, producing eleven coordinates for the egg profile, and exported

them into a .txt file (Fig. S1). Images before and after image processing were exported for visual inspection to ensure that no mistakes were made during the processing.

Verifying the absolute eggshell stiffness estimated by the finite element method (FEM)

In engineering, the stiffness of a load-bearing structure can be determined or estimated by three distinct approaches: experimental, numerical, and theoretical. Among them, the numerical approach such as FEM is particularly powerful and has been extensively used in engineering. However, FEM is a method of approximation and its accuracy must be verified by experiments and theories. In a previous study (Juang *et al.* 2017), we first conducted a side-by-side comparison of the key physical and mechanical properties of several representative species, and found that characterization of eggshell stiffness using egg images and numerical simulations was consistent with that obtained by experiments using mechanical compression tests (See Fig. 1 and Table S1 in Juang *et al.* 2017). Second, we did a side-by-side comparison of eggshell stiffness of several representative species between FEM and shell theory, and found that the deviation was < 3%, which reaffirmed the accuracy of FEM (See Table S2 in Juang *et al.* 2017).

C number as a proper measure of eggshell stiffness for interspecific comparisons

The absolute stiffness of eggshell— *K*—is improper for interspecific comparison of egg resistance to external loads from other eggs or incubating birds due to the confounding effect of egg size. For example, the absolute stiffness of a massive elephant bird egg (*Aepyornis maximus*, $K \approx 6.6\times10^{6}$ N m^{-1}) is much larger than that of a hummingbird egg (*Mellisuga minima*, $K \approx 1.1\times10^{4}$ N mm^{-1}); however, it is unclear whether an elephant bird egg, compared to a hummingbird egg, is more likely to survive the collision of its neighboring eggs (induced by the incubating bird or other environmental forces), as an elephant bird egg

is much heavier than a hummingbird egg and can cause more impact damage due to its higher momentum. To overcome this, Juang *et al.* (2017) developed a dimensionless metric, *C* number, to characterize eggs' stiffness with respect to egg mass and shape. Juang *et al.* (2017) estimated the *C* numbers of the elephant bird and hummingbird eggs to be 11,993 and 17,400, respectively. That is, the elephant bird egg showed a similar or slightly lower level of crash resistance than the hummingbird egg without the confounding effects of egg weight or geometry-induced rigidity. Therefore, the *C* number facilitates the comparison of eggshell stiffness across the avian phylogeny and even over the evolutionary history.

Theoretical analysis and experimental verification of the effect of simulated compression direction on estimated C numbers

To compare the *C* number estimated from the absolute stiffness (*K*) simulated along the horizontal axis with that along the vertical axis, we conducted the compression simulation test with the egg sample positioned horizontally, with its equator in contact with the two rigid plates (see Fig. S5b in Juang *et al.* 2017). The loading process was then approximated as indentation of a nonaxisymmetric convex shell at its equator, where the two principal curvatures $\kappa_1 = 1/b$ and $\kappa_2 = 1/a$ were in general not equal, where a and b were equatorial circular radius and polar radius, respectively. Thus, replacing $\kappa = 1/r$ by the mean curvature κ_M (Lazaru *et al.* 2012), we obtained $C_H \equiv \frac{K}{(\kappa_M/2)W} = \frac{K}{W}(2r_M) = \frac{2r_M}{\delta} = \frac{1}{\bar{\delta}}$, where *K* was the absolute shell stiffness, *W* the egg weight, δ the compression displacement, and $\bar{\delta} = \delta/2r$ the normalized displacement. The subscript *H* denoted "horizontal" compression, compared to *V* denoting "vertical" compression. In this case, the indentation was locally identical to the indentation of a spherical shell of radius $r_M = 1/\kappa_M = 2/(\kappa_1 + \kappa_2)$. Hence,

$C_H \equiv \frac{K}{(\kappa_M/2)W} = \frac{K}{W}\left(\frac{2AB}{A+B}\right)$, where the shape/size factor became $(2AB)/(A+B)$.

Similarly, for the horizontal loading at the equator, we substituted

$K_H = \left[2Et^2 \big/ \sqrt{3(1-\nu^2)}\right]\kappa_M$ into C_H, and obtained

$C_H \equiv \frac{K_H}{(\kappa_M/2)W} = \frac{1}{(\kappa_M/2)W}\frac{2Et^2}{\sqrt{3(1-\nu^2)}}\kappa_M = \frac{4}{\sqrt{3(1-\nu^2)}}\frac{Et^2}{W} = C_V$, where E and ν were respectively the Young's modulus and Poisson's ratio of the shell, and t was the shell thickness. Thus, the C number gave the identical result, $C \propto Et^2/W$, independent of the compression direction since the geometry-induced rigidity was removed.

A previous study conducted a comparison of experimental results of C_V and C_H for 36 species to show that C numbers in both directions were indeed highly similar (see Fig. S5e in Juang *et al.* 2017). The small difference was attributed to the fact that egg was not a perfect ellipsoid so some slight deviation in the simulation result was expected. In this study, we used the vertical C_v because it is relatively easy to construct the computer models without worrying about the problem of asymmetric convex shell, and C_V and C_H are good approximation.

Higher energy cost associated with higher C numbers

Although eggshell with high stiffness may prevent eggs from cracking, it is energetically costly. The stiffness of an ellipsoidal elastic shell is $K = \left[2Et^2 \big/ \sqrt{3(1-\nu^2)}\right]\kappa$, where E, t, and ν are the Young's modulus, the thickness, and the Poisson's ratio of the shell, respectively. κ is the local curvature of the shell at the loading point (the pole of eggshell) and thus determined by the shape and size of eggshell. E and v are largely constant according

to mechanical compression tests on 400 freshly laid intact eggs from 40 bird species, which belonged to 16 families and 11 orders (see Fig. 1h in Juang *et al.* 2017). Other things being equal, producing a stiffer egg (i.e., a larger *K* value) requires a thicker shell (i.e., a larger *t* value). Consequently, a higher *C* number ($C \equiv \frac{K}{W}\frac{A^2}{B}$), which controls the effects of egg size and weight, also corresponds to a thicker shell, which led to a higher *K* value; a thicker shell requires more calcium, which is a critical but limited resource for reproduction in birds (Tilgar *et al.* 2002), among other materials, and thus is energy-expensive. Thus, natural selection may not always favor eggs with higher *C* numbers.

Definitions of nest character categories

We used three nest characters—site, structure, and attachment—to categorize the nests of the 1,350 species based on the descriptions on the *Handbook of the Birds of the World Alive* (del Hoyo 2015), which is now the Birds of the World (https://birdsoftheworld.org/). Following the definitions in a previous study (Fang *et al.* 2018), we classified nests into six site categories: tree, non-tree vegetation, ground, cliff/bank, underground, and water (Fig. S2a); five structure categories: scrape, platform, cup, cavity, and dome (Fig. S2b); and four attachment categories: basal, lateral, horizontally forked, and pensile (Fig. S2c). Regarding nest structure, scrape nests were recorded when eggs were directly laid without a nest constructed. Platform nests were the nests having lining such as grass, sticks, or feathers on the bottom, loosely intertwined into a platform, with none or little shielding on the edge. Cup nests were ones shaped like a cup with an erected, surrounding rim that was too shallow to cover the adult birds. Dome nests referred to those nests able for adult birds staying inside without exposing themselves and often characterized by a narrow entrance. Cavity nests were ones in holes excavated by the parental birds or other animals or in natural caves.

Among nest sites, tree sites indicated the nests built on a tree. Non-tree vegetation sites included the nests built on bushes, bamboo, or herbaceous vegetation such as reeds and vines. Ground sites referred to those nests built on the ground (including nests that were built over short grass and stably sat on the ground) or to the case where eggs were directly laid on the ground. Cliff/Bank sites indicated that the nests were built on cliffs, river banks, or rocks. Underground sites indicated nests built in underground burrows. Water sites were the nests that were built by piling pebbles from the bottom of a water body to form an "island" and topping vegetation on the peak, or built on the elevated spots of sediment that emerge from the water, or set on (under)water plants and built above the water. For nest attachments, basally attached nests were those supported from their bottoms, including those stably located among interweaving branches or inside cavities. Laterally attached nests were those attaching their lateral part(s) to reeds, trees, or other objects except horizonal branches. Horizontally forked nests were supported by two or more horizontal tree or bush branches by their sides (not bottom). Pensile attachments indicated that the nests were hanged down from supporting objects and suspended in the air.

Supplementary Results

Evolutionary interdependence between nest characteristics and eggshell stiffness

Attachment (Figure 3A):

The correlated evolution model test showed strong support for a dependent model between nest attachment and C number over an independent model (Bayes Factor = 9.3). The attachment type of the ancestor of modern birds was more likely to be basal than non-basal. On the other hand, the similar probabilities estimated for the ancestor with different levels of C number suggested that ancestral birds did not tend to have higher or lower stiffness than their offspring. The results also showed that for birds using basal attachment, their C number

was more likely to transit from high values to low ones (q31 = 27.52) than vice versa (q13 = 20.78). For birds using non-basal attachment, their *C* number was more likely to transit from low values to high ones (q24 = 21.19) than vice versa (q42 = 16.33). The results were consistent with our finding that lower *C* numbers in extant birds using basal attachment than those using other attachment types (Fig. 2). Higher transition rates from non-basal attachment to basal attachment than vice versa in birds with either high or low *C* numbers might explain the prevalence of basal attachment in extant birds. The overall patterns regarding the influential roles of *C* number values and nest attachment types on each other imply that it is more likely that the evolution of *C* number depends on nest attachment types than vice versa.

Site (Figure 3B):

The correlated evolution model test showed strong support for a dependent model between nest site and *C* number over an independent model (Bayes Factor = 14). According to the dependent model, the low estimated probabilities of tree/non-tree vegetation nest sites for the root of the avian phylogeny suggested tree/non-tree-vegetation as derived traits relative to other nest sites. For birds using non-vegetation nest sites, their *C* number was more likely to transit from high to low values (q31 = 25.08) than vice versa (q13 = 10.19); for birds occupying tree/non-tree-vegetation nest sites, their *C* number was slightly more likely to change from low to high values (q24 = 27.59) than vice versa (q42 = 25.53). The results were consistent with our finding that higher *C* numbers in extant birds using tree or non-tree vegetation sites than those using other sites (Fig. 2). For birds with either high or low *C* numbers, they were more likely to transit from non-vegetation nest sites to tree/non-tree-vegetation sites than vice versa, although the transition rates were generally low (< 12). The differences in the transition rates may explain why more extant birds build nests on trees or

non-tree vegetations than those on other nest sites. The overall patterns suggested that nest sites are more likely to determine the evolution of *C* number than the other way around.

Structure (Figure 3C):

The correlated evolution model test showed strong support for a dependent model between nest structure and *C* number over an independent model (Bayes Factor = 15.6). The high estimated probabilities of scrape/platform nests for the root of the avian phylogeny suggested this type of nest structure as an ancestral trait against other types. For birds using scrape/platform nests, their *C* numbers were more likely to transit from high to low values ($q31 = 21.3$) than vice versa ($q13 = 9.27$); for bird using nests with other structure types, the transition rate ($q24 = 28.95$) from low to high *C* numbers was slightly higher than that of the opposite direction ($q42 = 26.96$). The results were consistent with our finding that lower *C* numbers in extant birds using scrape/platform nests than those using other structure types (Fig. 2). For birds with either high or low *C* numbers, they were more likely to transit from scrape/platform nests to other nest structure types than vice versa, although the transition rates were generally low (< 7). This may explain why there are slightly fewer extant avian species building scrape/platform nests than those building cup, dome or cavity nests. The overall patterns supported that the evolution of *C* number was more likely to be driven by than drive that of nest structure.

Supplementary Tables

Table S2. Terminology and nomenclature in eggshell stiffness estimation. The definitions of the terms and how they were determined are provided. FEM stands for the finite element method, a computer simulation method routinely used by engineers to predict the stiffness of a structure, e.g., bridges, buildings, vehicles, and airplanes.

Terms	Symbols	Definition	Determined by
The *C* number	C	$C \equiv \frac{K}{W}\frac{A^2}{B}$	Definition
Shell stiffness	K	The initial slope of the simulated load-displacement curve	FEM
Egg weight	W	The fresh egg weight	Schönwetter & Meise (1960)
Egg breath	A	The maximum lateral diameter of the egg	Schönwetter & Meise (1960); MVZ, UC Berkeley
Egg length	B	The maximum length of the shell	Schönwetter & Meise (1960); MVZ, UC Berkeley
Shell thickness	t	Shell thickness (without membrane)	Schönwetter & Meise (1960)
Young's modulus	E	Elastic constant used in FEM simulations	30 GPa (assumed, Juang *et al.* 2017)
Compressive force	F	The load applied to the egg	FEM
Displacement	δ	The deformation of the egg due to compressive force	FEM

Table S3. Summary of a single PGLS model for examining the effects of the three nest characters on *C* number among the studied species. Clutch size was included as an independent variable to account for its confounding effect.

	Coefficient	SE	t	P	Lambda
					0.413
Intercept	4.081	0.045	90.784	<0.001	
Lateral/Horizontal vs. Basal	0.023	0.019	1.180	0.238	
Pensile vs. Basal	0.045	0.025	1.756	0.079	
Tree vs. Others	0.030	0.011	2.675	0.008	
Non-tree vegetation vs. Others	0.049	0.012	4.126	<0.001	
Cavity vs. Scrape/Platform	0.058	0.013	4.372	<0.001	
Cup vs. Scrape/Platform	0.044	0.012	3.664	<0.001	
Dome vs. Scrape/Platform	0.097	0.017	5.891	<0.001	
Clutch Size	0.016	0.002	7.846	<0.001	

Table S4. Summary of the PGLS model for examining the interactive effect of nest attachment and site.

	Coefficient	SE	t	P	Lambda
					0.439
Intercept	4.117	0.047	88.021	<0.001	
Non-basal vs. Basal	0.049	0.022	2.264	0.024	
Non-tree vegetation vs. Others	0.050	0.010	5.101	<0.001	
Non-basal x Non-tree vegetation	0.029	0.030	0.967	0.334	
Clutch Size	0.018	0.002	8.370	<0.001	

Table S5. Quantile regressions on the median of *C* number values for the 1,350 bird species studied and their ancestors. The branch length from each extant or ancestral species to the root on the phylogenetic tree (node depth, indicating relative evolutionary time) was used as the independent variable in the regression models. A binary variable (Passerine) with 1 for passerine species and 0 for non-passerine species and its interaction with the node depth (Node depth x Passerine) are also included as independent variables. The *C* number values were log-transformed and node depths were standardized (i.e., centered and scaled) in the models.

**(A) Median (All)**	**Coefficient**	**SE**	**t**	**P**
Node depth	0.003	0.002	1.485	0.138
Intercept	4.228	0.003	1547.45	<0.001
**(B) Median (Passerines vs Non-passerines)**				
Node depth	-0.005	0.002	-2.759	0.006
Passerine	0.072	0.007	10.637	<0.001
Node depth x Passerine	-0.006	0.005	-1.327	0.185
Intercept	4.190	0.006	754.73	<0.001

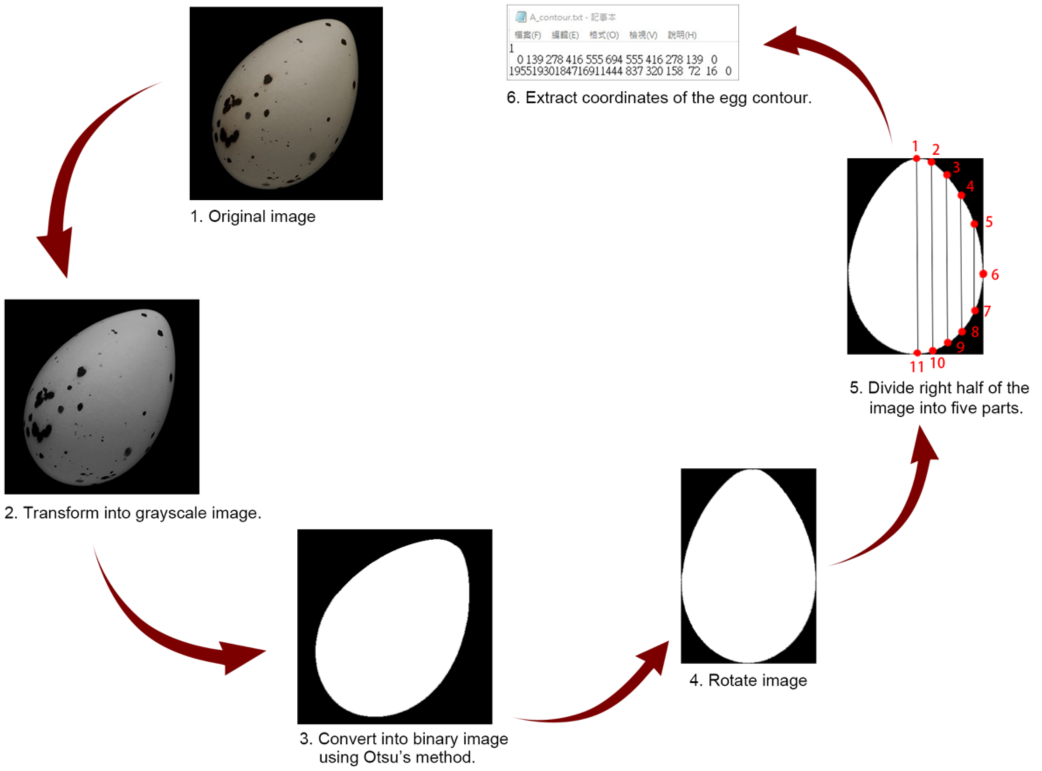

Figure S1. The image processing process.

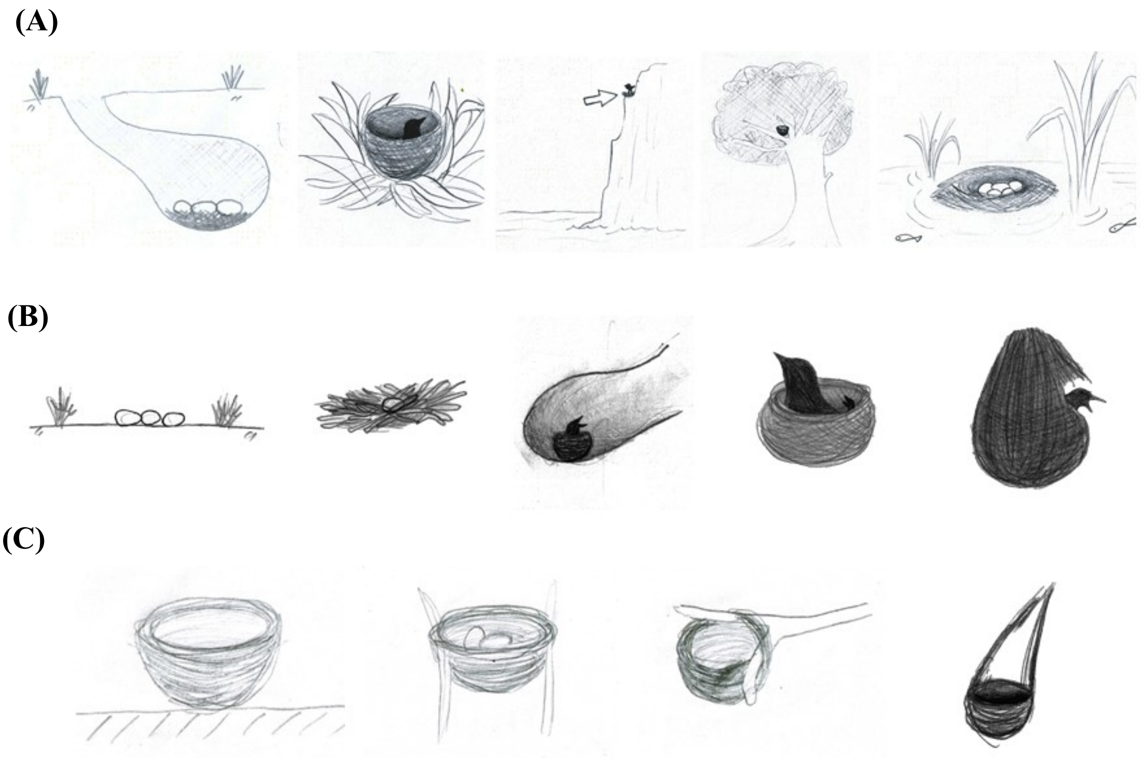

Figure S2. Demonstration of various nest sites, structures, and attachment. (Nest sketches credited to Shu-Han Tsao) (**A**) Nest site: (from left to right) underground, non-tree vegetation, cliff/bank, tree, water. (**B**) Nest structure: scrape, platform, cavity, cup, dome. (**C**) Nest attachment: base, lateral, horizontal, pensile.

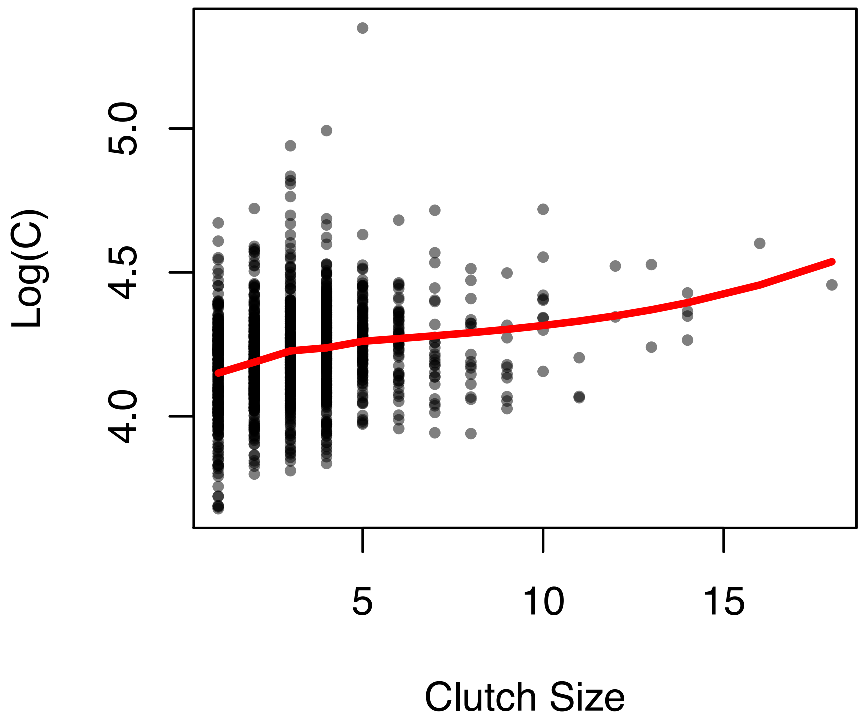

Figure S3. Relationship between clutch size and *C* number among the 1,350 bird species studied. The values of *C* number were log transformed. The red line shows the loess regression line fitted to the data.

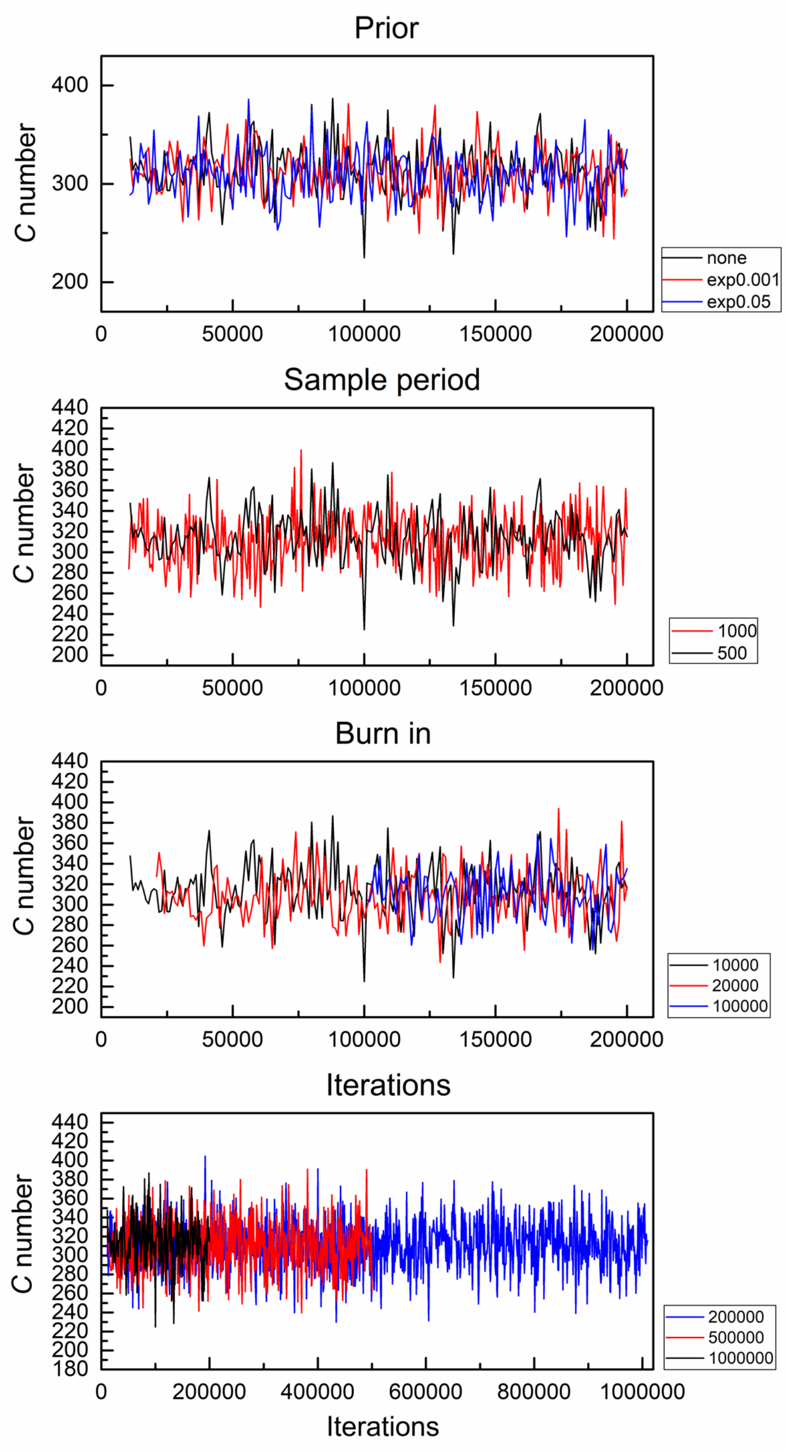

Figure S4. Convergence test results for BayesTraits analysis. Parameters included different priors, sample periods, burn in, and iterations.

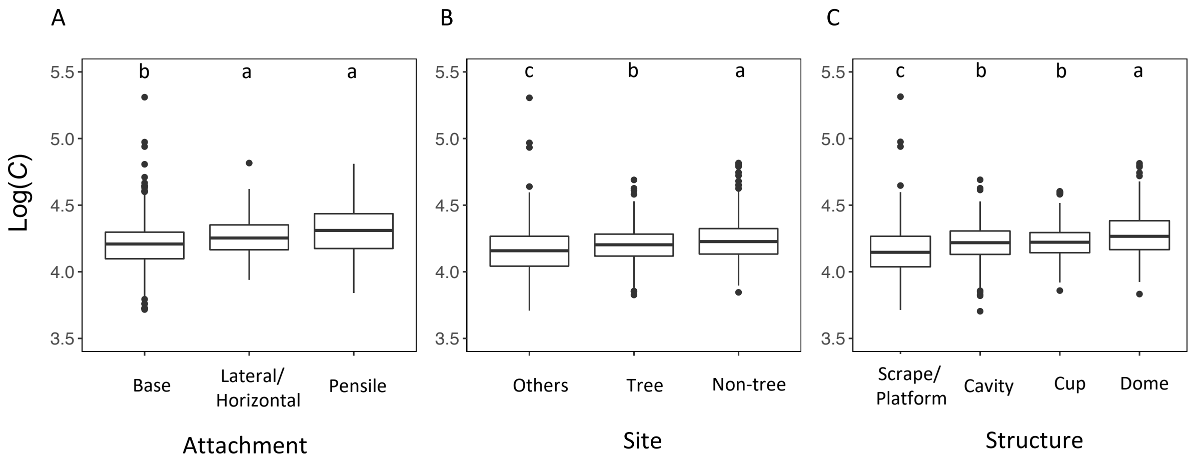

Figure S5. Variations in the eggshell *C* number values among birds using nests with different character types. Same as Fig. 2, but with a single PGLS model including the types of all three nest characters.

References

Burnham, K.P., Anderson, D.R., & Huyvaert, K.P. (2011). AIC model selection and multimodel inference in behaviorial ecology: some background, observations and comparisons. Behavioral Ecology and Sociobiology, 65, 23-25.

Dayton, C.M. (1998). Information criteria for the paired-comparisons problem. American Statistician, 52 144-151.

del Hoyo, J. (2015). *Handbook of the Birds of the World Alive*. Lynx Edicions.

Fang, Y.-T., Tuanmu, M.-N. & Hung, C.-M. (2018). Asynchronous evolution of interdependent nest characters across the avian phylogeny. *Nature Communications*, 9, 1-8.

Hauber, M.E. (2014). The Book of Eggs. University of Chicago Press.

Hothorn, T., Bretz, F. & Westfall, P. (2008). Simultaneous inference in general parametric models. Biometrical Journal: Journal of Mathematical Methods in Biosciences, 50, 346-363.

Jetz, W., Thomas, G.H., Joy, J.B., Hartmann, K. & Mooers, A.O. (2012). The global diversity of birds in space and time. Nature, 491, 444-448.

Jetz, W., Thomas, G.H., Joy, J.B., Redding, D.W., Hartmann, K. & Mooers, A.O. (2014). Global distribution and conservation of evolutionary distinctness in birds. Current Biology, 24, 919-930.

Juang, J.-Y., Chen, P.-Y., Yang, D.-C., Wu, S.-P., Yen, A. & Hsieh, H.-I. (2017). The avian egg exhibits general allometric invariances in mechanical design. *Scientific Reports*, 7, 1-11.

Lazarus, A., Florijn, H.C.B. & Reis, P.M. (2012). Geometry-Induced Rigidity in Nonspherical Pressurized Elastic Shells. *Physical Reviw Letters*, 109, 144301.

Pinheiro, J., Bates, D., DebRoy, S., Sarkar, D. & Team, R.C. (2007). Linear and nonlinear mixed effects models. R package version, 3, 1-89.

Popescu, A.-A., Huber, K.T. & Paradis, E. (2012). ape 3.0: New tools for distance-based phylogenetics and evolutionary analysis in R. Bioinformatics, 28, 1536-1537.

Revell, L.J. (2012). phytools: an R package for phylogenetic comparative biology (and other things). Methods in Ecology and Evolution, 3, 217-223.

Schönwetter, M. & Meise, W. (1960). *Handbuch der Oologie*. Akademie-Verlag Berlin, Germany.

Tilgar, V., Mänd, R. & Mägi, M. (2002). Calcium shortage as a constraint on reproduction in great tits Parus major: a field experiment. *Journal of Avian Biology*, 33, 407-413.
